# An Atlas of the Knee Joint Proteins and Their Role in Osteoarthritis Defined by Literature Mining

**DOI:** 10.1016/j.mcpro.2023.100606

**Published:** 2023-06-24

**Authors:** Rocío Paz-González, Lucía Lourido, Valentina Calamia, Patricia Fernández-Puente, Patricia Quaranta, Florencia Picchi, Francisco J. Blanco, Cristina Ruiz-Romero

**Affiliations:** 1 Grupo de Investigación de Reumatología (GIR) - Unidad de Proteómica, Instituto de Investigación Biomédica de A Coruña (INIBIC), Sergas, Complexo Hospitalario Universitario de A Coruña (CHUAC), A Coruña, Spain; 2 Departamento de Fisioterapia, Medicina y Ciencias Biomédicas, Grupo de Investigación de Reumatología y Salud (GIR-S), Centro Interdisciplinar de Química e Bioloxía (CICA), Universidade da Coruña (UDC), A Coruña, Spain; 3 Centro de Investigación Biomédica en Red de Bioingeniería, Biomateriales y Nanomedicina (CIBER-BBN), Madrid, Spain

**Keywords:** knee, osteoarthritis, proteomics, cartilage, synovial, subchondral bone, meniscus, cruciate ligament, human proteome project, chondrocytes, synoviocytes, nitric oxide, apoptosis, mitochondria

## Abstract

Osteoarthritis (OA) is the most prevalent rheumatic pathology. However, OA is not simply a process of wear and tear affecting articular cartilage but rather a disease of the entire joint. One of the most common locations of OA is the knee. Knee tissues have been studied using molecular strategies, generating a large amount of complex data. As one of the goals of the Rheumatic and Autoimmune Diseases initiative of the Human Proteome Project, we applied a text-mining strategy to publicly available literature to collect relevant information and generate a systematically organized overview of the proteins most closely related to the different knee components. To this end, the PubPular literature-mining software was employed to identify protein-topic relationships and extract the most frequently cited proteins associated with the different knee joint components and OA. The text-mining approach searched over eight million articles in PubMed up to November 2022. Proteins associated with the six most representative knee components (articular cartilage, subchondral bone, synovial membrane, synovial fluid, meniscus, and cruciate ligament) were retrieved and ranked by their relevance to the tissue and OA. Gene ontology analyses showed the biological functions of these proteins. This study provided a systematic and prioritized description of knee-component proteins most frequently cited as associated with OA. The study also explored the relationship of these proteins to OA and identified the processes most relevant to proper knee function and OA pathophysiology.

## Highlights

• Osteoarthritis is a very prevalent disease, causing a huge socioeconomic impact.
• Proteomic information on knee tissues has not been systematically analyzed.
• Literature mining analysis provides an atlas of the knee as an organ.
• Specific pathway alterations in the knee are key in osteoarthritis pathophysiology.

## Graphical Abstract

**Figure:**
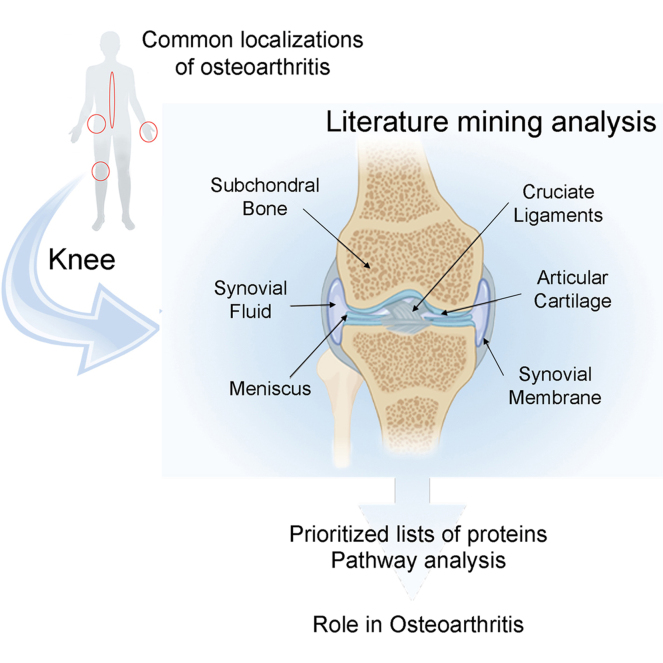

## In Brief

Information gathered by the molecular analysis of knee joint tissues is essential to improve the management of knee osteoarthritis, the most prevalent rheumatic pathology. Pursuing one of the goals of the Human Proteome Project, we applied a systematic strategy based on literature mining to define a protein atlas of this organ, including the prioritization of the proteins most cited in its most representative tissues. Pathway analysis on these proteins depicted the most relevant processes contributing to OA pathophysiology.

Osteoarthritis (OA) is a chronic rheumatic disease characterized by a functional limitation of joint mobility. The prevalence of OA has doubled in the last two decades, currently affecting more than 500 million people worldwide (1). OA thus has a significant socioeconomic impact, ranking this pathology as one of the first causes of disability in elderly people and resulting in economic costs ranging from 1% to 2.5% of the gross national product (2). OA primarily affects weight-bearing joints, such as those of the knee, hip, and spine, but it can also affect the hand. OA of the knee (KOA) is one of the most common manifestations and accounts for 10% of the worldwide population, making it an important focus from a public health perspective (3).

The knee joint is an organ constituted of several highly specialized tissues organized in a very precise manner to facilitate proper joint function (4). Therefore, failure in a single component compromises joint integrity and can trigger KOA. As such, OA is recognized as a whole-joint disorder in which the first step involves molecular changes. These changes are followed by structural damage, mainly cartilage loss but also synovial inflammation, subchondral bone remodeling, and ligament and meniscus alterations (5). Remarkably, despite the large number of proteomic and other molecular studies carried out in the last decade, the massive amount of data generated in this area remain disorganized and lack systematization. For example, the Human Protein Atlas repository contains no information regarding any of the knee tissues (6). Moreover, a very recent large-scale, single-cell transcriptomic analysis of 192 different cell types did not include any cartilage, synovium, or bone-related cells (7). Consequently, there is an urgent need for efficient management and organization of this information to maximize the impact of proteomic research.

The Human Proteome Project (HPP) was launched in 2010 to provide new insights into the human proteome. The Biology and Disease-driven (B/D)-HPP is one of the central initiatives of this project, and it was created with the aim of developing targeted and high-throughput proteomics analyses to expand knowledge regarding human proteins. One research branch of the (B/D)-HPP is the Rheumatic and Autoimmune Diseases (RAD) initiative of the Human Proteome Project (RAD-HPP). This resource is focused on solving unmet clinical needs related to the physiopathology, prognosis, and treatment of rheumatic disorders, thereby promoting the progression from proteomics-based discovery to translational research in clinical routines. The goals of the RAD-HPP include proteomic characterization of human joint tissues and the assembly of prioritized lists of so-called “popular” (or prioritized/high priority) proteins that may be clinically relevant in rheumatic disorders using literature mining platforms to systematically extract information (8, 9).

Altogether, more than 32 million published articles are available in PubMed, and more than eight million of these articles are related to proteins. This overload of information highlights the need to summarize and organize protein-related findings to discover new trends or focus research efforts on high-priority proteins within a given topic of interest. Text-mining strategies provide tools to extract meaningful information from the scientific literature (10). A text-mining approach was recently employed to identify the most-cited or “popular” proteins closely related to rheumatic and musculoskeletal diseases such as rheumatoid arthritis, spondyloarthropathies, systemic autoimmune connective tissue disorders, and OA (11).

In the present study, we performed a large-scale literature search employing data-mining tools to prepare prioritized lists of proteins referenced in the literature that are associated with the most-representative knee components and explore their relationship to KOA. Using this approach, we generated a systematic and organized overview of the proteins most closely related to the knee joint based on references in PubMed. The resulting data will facilitate the development of targeted studies in the field and enable the characterization of protein biomarkers that can be used in therapeutics development, treatment monitoring, and as a basis for future precision medicine strategies (12).

## Experimental Procedures

### Literature Mining Analysis to Create a Disease Map of OA

The latest available version of PubPular (https://heart.shinyapps.io/PubPular/) was used to identify protein-topic relationships and extract the most frequently cited proteins associated with the different knee components and OA localization up to November 2022. This method was based on calculating the semantic similarity between a protein and a query term in the literature. Its algorithm determines the relevance of a protein in a topic of interest by calculating the weighted co-publication distance (WCD) score, which considers the immediacy and impact of individual publications to adjust the contributions of single publications to popularity scores (11, 13).

The following keywords were entered for searching in PubPular to obtain an overall grouping of proteins specifically associated with the most common localizations of OA: “knee osteoarthritis”, “hip osteoarthritis”, “hand osteoarthritis”, and “spinal osteoarthritis”. Next, to identify proteins associated with the different knee tissues, the terms “articular cartilage”, “subchondral bone”, “synovial fluid”, “synovial membrane/tissue”, “meniscus”, and “cruciate ligament” were employed. The bibliometric analysis was performed on the totality of publications curated in PubMed without date restrictions. Proteins identified for each keyword were prioritized based on their WCD score from best to worst and identified using their UniProt accession number.

### Pathway and Gene Ontology Analysis

Further analyses were performed on the lists of proteins generated by PubPular using the STRING v 11.5 tool (https://string-db.org/) in order to visualize and explore interactions between the top 100 proteins identified within each topic (14). Only interactions scoring above the highest confidence (required score >0.9) and a stringency of 1% false discovery rate (FDR) were included in the networks (supplemental Figs. S1–S3). Disconnected nodes in the network were hidden.

Additionally, Gene Ontology (GO) pathway enrichment analyses were performed using the retrieved protein lists to identify the significantly associated biological processes, which were calculated with adjustment of the FDR using the Benjamini-Hochberg method. The results are presented as semantic similarity scatterplots generated using the REVIGO web server with the provided R script and employing the ggplot2 and scales R packages (15). Using this tool, the scatterplots showed GO terms as circles arranged such that those that were most similar in semantic spaces X and Y were placed nearest to each other. The input GO term list was extracted from the previous STRING analysis filtered by the FDR, and the most-significant terms were labeled with their descriptions in the plots. Finally, Venn diagrams were prepared using the InteractiVenn tool (http://www.interactivenn.net/index2.html) to examine similarities at the protein level among the different joint tissues.

## Results and Discussion

### The Knee as a Source of Prioritized Proteins for Drug Development and Identification of OA Biomarkers

**Fig. 1:**
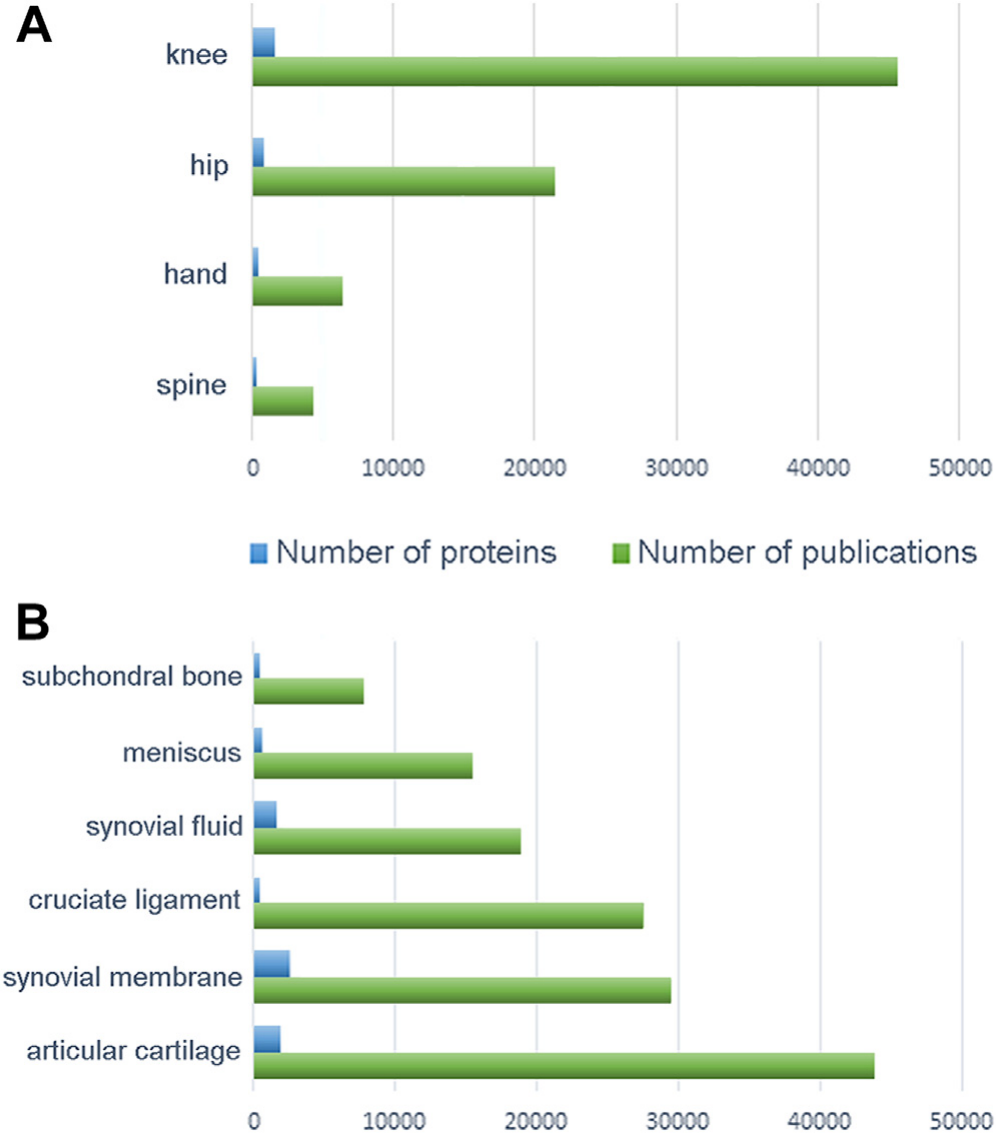
**Literature mining analysis.** Number of proteins (*blue*) and number of publications (*green*) identified in this work by literature mining in (*A*) the most common localizations of OA and (*B*) the most representative knee tissues.

**Fig. 2:**
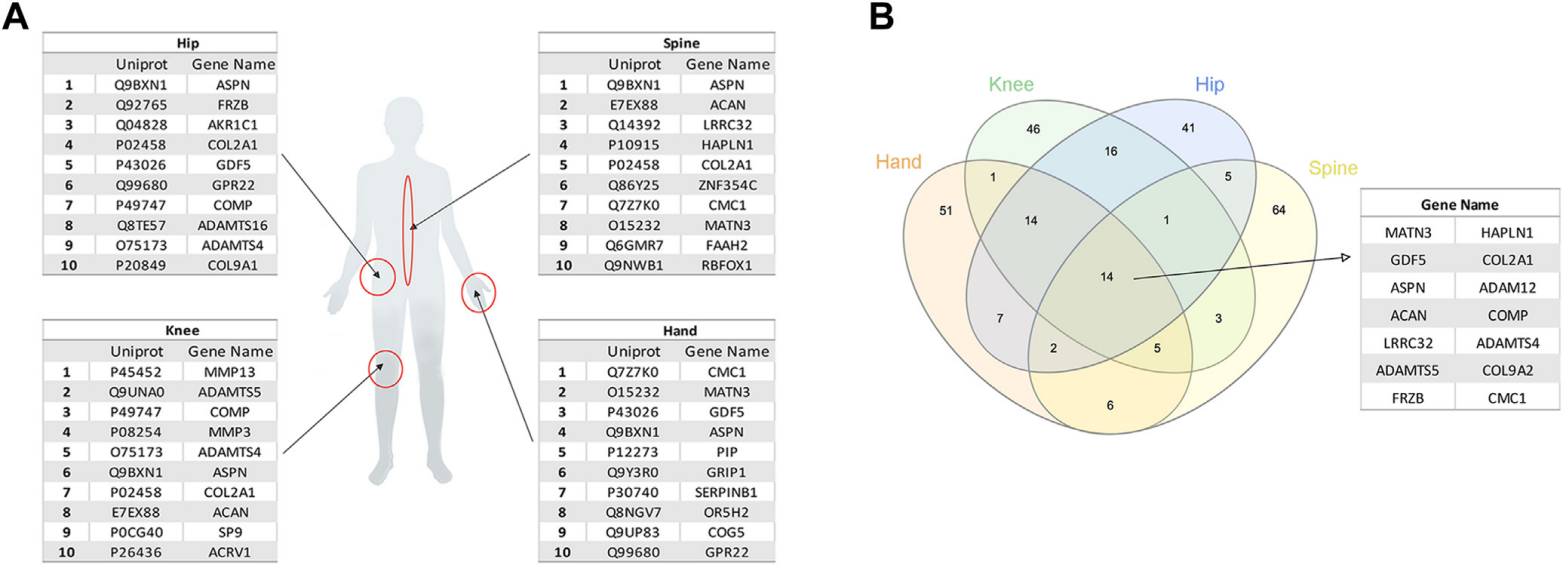
**Proteomic map of the most common OA localizations.***A*, *top* ten most cited proteins in the most common joints affected in OA, according to their WCD score using the PubPular tool. *B*, overlap of the top 100 proteins identified in each localization.

The knee is by far the most extensively studied site of OA localization, with 45,624 publications citing 1676 different proteins (Fig. 1*A*). Accordingly, most studies of knee proteins have been prompted by OA. Regarding other joints also frequently affected by OA, the hip was the second-most-common site of localization to be analyzed, with approximately half as many papers as have been published for the knee. The hip was followed at a considerable distance by the hand and spine, further underscoring the suitability of studying the knee to identify OA protein biomarkers. The top ten proteins identified in each of these joints are illustrated in Figure 2*A*, and a complete list detailing the top 100 proteins is shown in supplemental Table S1.

Overall, as shown in Figure 2*B*, only 14 proteins appeared among the top 100 in all OA localization sites, but these proteins are potential high-priority markers of OA, independent of localization. Although many of these proteins have been extensively studied in the field of OA research, other proteins, such as FZRB and LRRC32, have interesting value as targets for further research. Interestingly, FZRB (secreted frizzled-related protein 3, or SFRP3) is an antagonist of the Wnt8 signaling pathway that regulates chondrocyte maturation and joint homeostasis (16). It was recently shown that mechanical stress reduces SFRP expression and promotes temporomandibular joint OA *via* Wnt (17). However, an alternative role for sFRP family proteins that are independent of the Wnt pathway was also recently reported but remains to be further explored, as this role could potentially expand their potential therapeutic uses (18). By comparison, LRRC32 is a key regulator of transforming growth factor beta activation (19). Although some transcriptomic and genome-wide association studies have implicated the LRRC32 gene as playing a role in OA (20, 21), to our knowledge, no studies have evaluated this potential association at the protein level or its putative functional significance.

Proteins associated with the different knee compartments were then explored, together with the number of publications screened in each case (Fig. 1*B*). As shown in the figure, articular cartilage is the most-studied joint tissue, with 43,815 papers identified by literature mining that describe 2052 proteins. The second most-studied compartment was the synovial tissue. In contrast, the subchondral bone is the least-cited compartment, indicating that much of this tissue and its role in disease pathogenesis remains to be studied.

### The Articular Cartilage Proteome

**Fig. 3:**
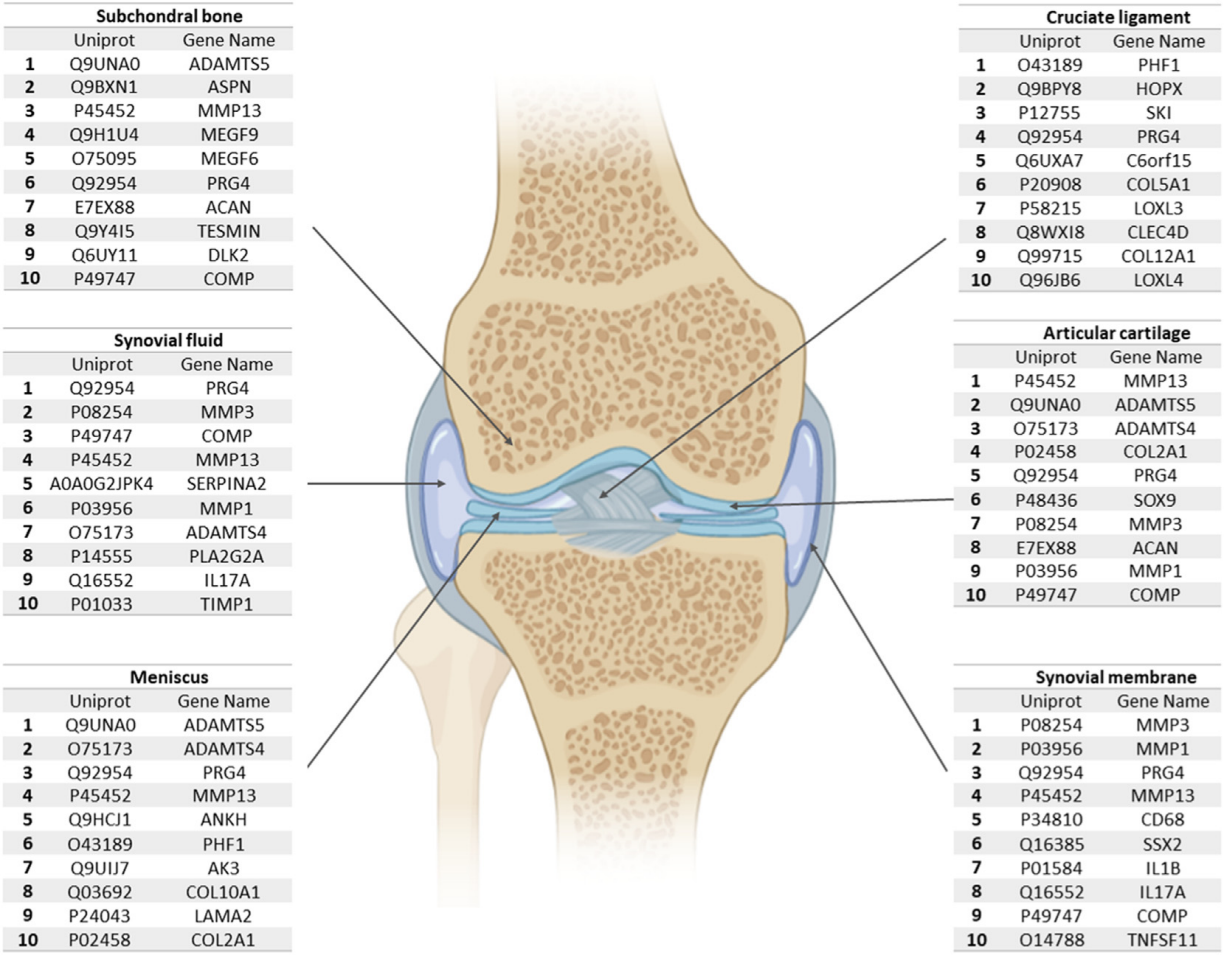
**Proteomic map of the different knee components.***Top* ten proteins in each of the six most representative components of the knee joint, according to their WCD score using the PubPular tool.

OA primarily affects articular cartilage. A major research goal in this area has been to discover molecular biomarkers that reflect changes in cartilage composition, especially in early disease stages (22). Articular cartilage consists of a small number of chondrocytes and extracellular matrix (ECM) supported by water and structural components, primarily collagen and proteoglycans (23). As shown in Figure 3, the top ten proteins identified in this tissue included collagen alpha-1 (II) chain (COL2a1) and proteoglycan 4 (PRG4), a chondrocyte-secreted glycoprotein involved in the lubrication of the boundary between joint surfaces. The top ten proteins also included proteinases responsible for ECM destruction, a key factor in OA progression. The proteinases responsible for the cleavage of collagen and proteoglycans from the ECM include matrix metalloproteinases (MMPs) and disintegrin-metalloproteinases with thrombospondin motifs (ADAMTS) (24). The first mediators in the degradation process are ADAMTS-4 and ADAMTS-5. MMP-13 and other MMPs then continue to degrade collagen II and aggrecan, the most abundant proteoglycan in the ECM (25).

**Fig. 4:**
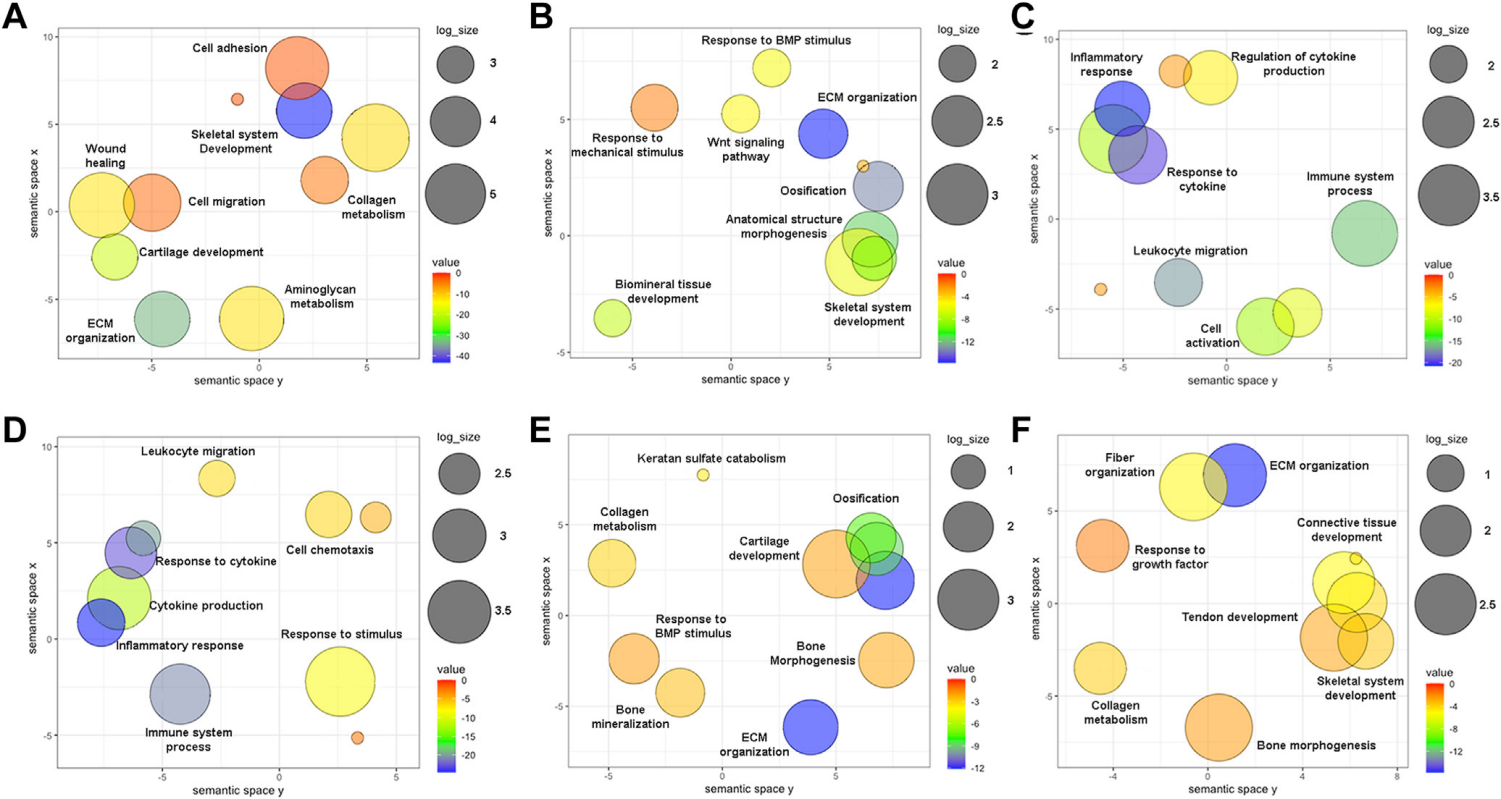
**Gene ontology analysis of the knee proteome defined by literature mining.** Visualization by semantic similarity scatterplots of the GO terms that are enriched in the six components of the knee that have been analyzed in this study (FDR 0.01). *A*, articular cartilage, (*B*) subchondral bone, (*C*) synovial membrane, (*D*) synovial fluid, (*E*) meniscus, and (*F*) cruciate ligaments. The results are shown using the REVIGO web server (15). The significance obtained from the enrichment analysis is shown using a gradient color palette (log10 *p*-value), and the sizes of the plotted circles are scaled by the GO term frequency.

Figure 4*A* illustrates the results of GO analysis to identify the key biological processes mediated by the proteins in this tissue most cited in the literature. The pathways related to these processes, which are primarily associated with ECM organization and the development of the skeletal system, are illustrated in supplemental Fig. S1*A*.

### The Subchondral Bone Proteome

Despite the key role of subchondral bone in joint pathology, it is the least-studied knee component, with only 7684 papers and 504 proteins identified through literature mining (Fig. 1*B*). As previously stated, the central feature of OA is joint cartilage breakdown, which is radiologically visible as narrowing of the joint space. However, features noted on diagnostic imaging also include subchondral bone alterations such as bone sclerosis, formation of osteophytes and bone cysts, and bone attrition (26). Crosstalk between chondrocytes and bone cells (osteoclasts, osteoblasts, and osteocytes) involved in the regulation of joint homeostasis has been clearly established (27, 28). However, only recently has the crucial role of subchondral bone in the early stages of OA been revealed. This tissue lies subjacent to the articular cartilage and provides the latter with mechanical and nutritional support. Disturbances in the subchondral bone microenvironment lead to osteoblast dysfunction and poor mineralization, thereby increasing cytokine synthesis and benefiting a pro-catabolic chondrocyte phenotype (29, 30). This triggers cartilage matrix degradation through the induction of MMP-13 (31, 32), an enzyme identified along with ADAMTS-5 among the top ten most relevant proteins in the subchondral bone (Fig. 3) (33). Osteoblasts isolated from the sclerotic zone of OA subchondral bone significantly inhibit aggrecan production and significantly increase MMP-3 and MMP-13 synthesis (34). This highlights how articular cartilage integrity depends on proper subchondral bone function at the cellular, structural, and functional levels. GO analysis (Fig. 4*B*) and pathway visualization (supplemental Fig. S1*B*) revealed that the ECM organization and ossification processes are the most representative pathways related to this knee component.

Bone is a dynamic tissue that is continuously remodeled *via* two mechanisms: bone resorption, mediated by osteoclasts, and new bone formation, mediated by osteoblasts. Together, these processes maintain the equilibrium of the skeletal system in terms of size and shape (35). Bone remodeling is mediated by osteocytes, the third cell type in bone (36). Transforming growth factor-beta (TGF-β) is one of the most important growth factors involved in chondrogenesis and the control of MMPs expression. Asporin (ASP), which inhibits TGF-β, is one of the top ten proteins in the subchondral bone (Fig. 3). ASP regulates osteoblast-driven collagen biomineralization activity and was shown to be upregulated in articular cartilage and osteoblasts isolated from OA patients (37). Emerging data regarding bone-cartilage crosstalk and subchondral bone metabolism have provided new targets for OA management. Approaches that target the subchondral microenvironment or reduce angiogenesis and nerve formation from the subchondral bone into the articular cartilage could have therapeutic potential (38). As an extracellular and tissue-specific protein, ASP represents a promising target (39).

Angiogenesis plays essential roles in bone growth and remodeling and in supporting avascular articular cartilage. Abnormal angiogenesis occurs at the cartilage-subchondral bone interface in OA, and this process is mediated by the overproduction of angiogenic factors such as vascular endothelial growth factor, receptor activator of NF-κB ligand, and epidermal growth factor (EGF)-like family member. In addition to their role in angiogenesis, some EGF-like family members stimulate epidermal growth and keratinization and facilitate communication between bone cells and endothelial cells, which is required for the proper balancing of bone remodeling processes. Expression of EGF-like family members in the bone microenvironment was recently identified, but the specific functions of these proteins remain unclear (35, 38). As shown in Figure 3, literature mining identified two EGF-like family members preferentially expressed in osteoblasts as the most frequently cited proteins in subchondral bone: multiple epidermal growth factor-like domains protein 6 (MEGF6), better known by its alternative name, epidermal growth factor-like protein 3 (EGFL3), and MEGF9/EGFL5 (35).

### The Synovial Tissue Proteome

The synovium is a soft tissue comprising two layers: an outer layer (or subintima), which is relatively acellular but rich in type I collagen and blood vessels, and an inner layer (or intima), which consists of one to three layers of fibroblast-like synoviocytes and macrophages (40). Research involving synovial tissue has expanded tremendously in recent years, mainly due to advances making visualization easier and improvements in the reliability of synovial biopsies through arthroscopic and ultrasonographic technology (41). As of November 2022, a total of 29,468 papers were identified by literature mining, and these papers describe 2572 proteins associated with the synovial membrane (Fig. 1*B*). The vast majority of the top 100 identified proteins related to this tissue are involved in inflammatory and immune processes that in turn mediate destruction of the structural components of cartilage (Fig. 4*C* and supplemental Fig. S2*A*). This highlights the close relationship between synovial tissue and inflammatory responses.

*In vitro* evidence has shown that synovial fibroblasts control ion transport for nutrient exchange between the synovial fluid and the synovial membrane. These cells also maintain the joint structure by secreting various ECM components, such as type II, IV, V, and VI collagens, proteoglycans, fibronectin, laminin and tenascin, and proteinases such as MMPs and cathepsins (42). Molecules released into the synovial fluid from degraded cartilage likely initiate synovial inflammation in OA, which is characterized by synovial lining hyperplasia, sublining fibrosis, and stromal vascularization. Synovitis is a common feature of OA and is associated with clinical symptoms and progressive joint failure in a subgroup of patients. Evidence supporting the inflammatory-OA phenotype is provided by the good effect of anti-inflammatory therapies such as non-steroidal anti-inflammatory drugs and intra-articular corticosteroid injections (43) at relieving OA pain.

Three of the 10 top-ranked proteins in the synovial membrane (Fig. 3) are MMPs. MMP-1, -3, and -13 are the most frequently cited according to our PubPular search, and all of these proteins play a role in collagen degradation at different levels. Synovitis may accelerate the catabolism of articular cartilage in OA *via* the production of large amounts of MMPs. Indeed, expression of the MMP-3 and MMP-9 genes is upregulated in swollen areas of OA synovium compared with normal areas (44). Expression of these MMPs in the synovium is positively correlated with the severity of OA (45).

PRG4 was first identified in synovial fluid as a specific product of the synthesis activity of chondrocytes in the superficial zone of articular cartilage, but it has also been detected in the synovial membrane, tendons, ligaments, discs, and menisci (46). PRG4 is commonly referred to as lubricin and plays an important role in maintaining synovial macrophage homeostasis, but a lack of PRG4 expression leads to an increase in the number of pro-inflammatory macrophages in the synovium (47). Finally, the CD48 antigen (CD48), also known as the B-lymphocyte activation marker (BLAST1) or signaling lymphocytic activation molecule 2 (SLAMF2), was ranked fifth on the most-cited proteins list. The number of subliming-associated CD68 macrophages is used as a parameter to distinguish rheumatoid arthritis (RA) from OA and has been described as one of the best RA activity markers (48).

### The Synovial Fluid Proteome

The synovium encapsulates the synovial fluid, a highly viscous liquid rich in hyaluronic acid and lubricin. Synovial fluid results from the ultrafiltration of blood and acts as a lubricant for articular cartilage during movement and as a nutrient source through diffusion to surrounding structures (49). Specifically, the PubPular software identified 18,906 papers describing 1665 proteins associated with synovial fluid (Fig. 1*B*).

The synovial fluid is the most useful biofluid for investigating changes in the joint environment, given its direct and close association with the different knee tissues (45). Changes in the synovium may be observed indirectly in the synovial fluid, as their close relationship is translated through common pathways (Fig. 4*D* and supplemental Fig. S2*B*) and a 55% overlap among the top 100 most-cited proteins (supplemental Fig. S2*C*). Indeed, MMP-3, MMP-13, MMP-1, ILA17A, and PRG4 are shared among the top ten most-cited proteins associated with the synovial fluid and synovial membrane.

Cartilage oligomeric matrix protein (COMP) is another extensively studied protein. COMP is an ECM glycoprotein originally isolated from cartilage but later shown to be expressed in a wide variety of joint tissues, including the synovium. COMP plays roles in numerous processes and pathologies, such as chondrocyte proliferation, thrombin inhibition, mechanical stress resistance, chondrodysplasias, cancer, and cardiomyopathies. Despite controversy regarding the role of COMP in OA, high levels of COMP locally produced in the joint are correlated with early-stage OA and RA as a result of cartilage degeneration (50). Many authors have suggested that the elevation of COMP levels in serum and synovial fluid is a useful diagnostic biomarker in OA (51). However, it remains unclear whether changes in COMP levels are sufficiently sensitive for evaluating KOA.

Finally, serpins are a superfamily of serine proteinase inhibitors with recognized roles in blood coagulation, embryonic development, and ECM turnover *via* activation of MMPs and direct proteolysis of the ECM. Serpins are inactivated in OA-affected joints, which promotes cartilage breakdown through the increased destructive activity of serine proteinases (52). The most-studied member of this family, SERPINA1, is the major inhibitor of neutrophil elastase, a well-described proteoglycan-degrading enzyme and potent activator of MMP-13, which is associated with inflammatory arthritis. Recent evidence suggests a potential role for SERPINA1 in OA (53), considering that lower levels of SERPINA1 have been observed in OA synovial fluid compared with non-OA controls. The sequence of SERPINA1 is highly homologous to that of SERPINA2, which was originally thought to be a pseudogene but was identified in this study among the top ten proteins in synovial fluid. Current evidence suggests that SERPINA2 is an active gene encoding an endoplasmic reticulum protein with activity divergent from that of SERPINA1, but no relationship with synovial fluid has been reported (54). A potential explanation for this result arises from the confusing nomenclature regarding these proteins, which might be also responsible for the previously underappreciated role of serine proteinases in OA (52).

### The Meniscus Proteome

The menisci are two wedge-shaped semi-circular fibrocartilage structures located between the surfaces of the femur and tibia in the medial and lateral compartments of the joint. They play a key role in load transmission, shock absorption, and joint stability. The major components of the meniscus matrix are water, collagen type I (98%), and proteoglycans (55). Despite the importance of the meniscus in knee function, it is the second least-studied tissue of the joint, with only 15,402 papers identified involving 596 associated proteins (Fig. 1*B*).

The relationship between OA and the meniscus is complex, but it is clear that the risk of the disease increases if the meniscus is damaged by injury or degenerative processes (56, 57). The principal biological alterations in the meniscus are calcifications and ECM degradation. Basic calcium phosphate crystals and calcium pyrophosphate dihydrate crystals are found in the knee joint fluid up to 65% of patients with OA (58). These crystals modify the biomechanical properties of the meniscus, fostering the production of inflammatory cytokines and matrix-degrading enzymes such as ADAMTS-5 and MMP-13. These enzymes, along with ADAMTS-4, are involved in ECM organization.

Among the proteins involved in the meniscal biomineralization process, pyrophosphatase/phosphodiesterase one and ankylosis progressive homolog (ANKH) are upregulated in OA meniscal cells. The function of ANKH, as one of the top five proteins most studied in the meniscus, involves the transport of inorganic pyrophosphate, a mediator in the mineralization process (59). As revealed by GO analysis, the top 100 proteins identified in this tissue are related to bone processes, ECM organization, and cartilage development (Fig. 4*E*). Furthermore, reorganization of the ECM and development of the skeletal system were identified as the most relevant pathways in this tissue (supplemental Fig. S3*A*).

### The Cruciate Ligament Proteome

Ligaments are collagenous connective tissues that join bone with bone by fixing areas called entheses. In the knee, the anterior and posterior cruciate ligaments connect the intercondylar areas between the femur and tibia. Intra-articular ligaments impart stability and balance loading to the joint. Injury to the anterior cruciate ligament (ACL) is the most common traumatic knee injury and increases the risk of developing posttraumatic OA, regardless of surgical reconstruction status (60, 61, 62). Cruciate ligaments are tissues with poor blood supply and are composed of isolated fibroblasts responsible for the synthesis of abundant ECM. This is reflected in supplemental Fig. S3*B*, which shows that ECM organization is the most studied pathway in this tissue. This ECM is composed primarily of water and type I collagen, with a proteoglycan content of <1% (60). GO analysis (Fig. 4*F*) showed the enrichment of processes related to the development of joint tissues.

The cruciate ligament is ranked as the third most studied tissue in the knee, with 27,559 papers retrieved by literature mining. However, only 563 proteins were identified in these studies (Fig. 1*B*). This discrepancy could be related to the heavy focus of ligament research on surgical methods, epidemiology, and rehabilitation of ACL injury (63). Considering the above, the most-cited proteins identified by literature mining do not appear as reliable candidates for further studies. Indeed, PRG4, a well-characterized protein related to the cruciate ligament, was identified using PubPular as among the top ten most-cited proteins (Fig. 3). Being the principal lubricating molecule in diarthrodial joints, PRG4 is down-regulated after ACL injuries by the pro-inflammatory mediators IL-1β and TNF-α, which compromises joint lubrication (64). Furthermore, although no effective treatments or surgical approaches are available to prevent the development of OA after an ACL injury, intra-articular supplementation with lubricin seems to mitigate the effects (65).

It seems some of the top ten proteins identified by PubPular as part of the cruciate ligament proteome were included as a consequence of misunderstanding or text-mining errors. The homeodomain-only protein (HOPX) is an unusually small protein that modulates target gene transcription, and some studies have linked HOPX with the regulation of skeletal muscle differentiation. However, HOPX is also related to ligaments, as the “Hop test” is a functional test universally used to assess the potential to return to sports activities after ACL reconstruction (66). Another example of curious misunderstanding is the inclusion of the Ski oncogene (SKI), considering that Alpine ski racing is associated with a high ACL injury rate; however, not a single study has related the ACL with SKI protein (67). Finally, the uncharacterized protein C6orf15 was identified among the top ten most-cited proteins (Fig. 2), also despite no clear evidence in the papers. We observed that the alternative name for C6orf15 is STG, and we, therefore, hypothesize in this case that the relationship between the ligament and STG is not really related to a protein but rather to the method for ACL reconstruction known as the semitendinosus and gracilis (STG) technique (68).

Finally, the overlap in the findings obtained by literature mining regarding the six knee components was explored (supplemental Fig. S4). Altogether, only eight proteins (MMP-1, -3, and -13, ADAMTS-4 and -5, ACAN, PRG4, and COMP) were identified among the top 100 in all knee components analyzed. This emphasizes the usefulness of individual analyses of the different components of a specific organ to obtain a more comprehensive overview of the pathways and processes mediating its normal or pathological functions.

### Role of the Knee Joint Proteome in OA

OA is a major public health concern and is ranked among the leading causes of chronic disability in people older than 50 years. Unfortunately, no accepted biomarkers considered surrogate endpoints that could be monitored for regulatory approval in OA trials have been identified. Despite several studies published in this area, no single biomarker stands out as the gold standard, and none even reach the validation phases for clinical use (69); overall, biomarkers for clinical practice are still an unmet need in OA.

The molecular biomarkers most studied in OA are COMP in serum and urinary cross-linked C-terminal telopeptide of type II collagen (uCTX-II) in urine (70). CTX-II, a fragment of COL2A1, is a potential biomarker proposed for early OA, and it was identified among the top ten most-cited proteins in the articular cartilage and meniscus (71). On the other hand, COMP was also ranked among the top ten most-cited proteins of the knee. This protein is highly abundant in cartilage and to a lesser degree in other knee tissues as well, such as the tendons and synovial membrane. Many studies have reported elevated COMP levels in primary OA and after knee injury. Studies of large and well-characterized OA cohorts have associated serum levels of COMP with the main features of structural damage in OA as well as with joint pain (72). However, the major drawback of COMP as a biomarker for OA is a lack of specificity, as elevated levels have also been reported in other chronic diseases (73). Apart from these two “classical” OA biomarkers, the plasma level of cartilage acidic protein 1 (CRTAC1) was recently suggested as a promising candidate for the early diagnosis of knee and hip OA (74). Although CRTAC1 was not identified among the top ten most-cited proteins in any knee tissue, it was included among the top 100 in the articular cartilage (ranked 61), meniscus (ranked 37), synovial fluid (ranked 38), and synovial membrane (ranked 85).

In addition to the biomarker candidates described above, roles in OA for the majority of the top ten most-cited proteins identified in the knee compartments have been reported. Table 1 shows the modulation of these proteins in different sample types. Many of the proteins have demonstrated potential clinical relevance for treating KOA or usefulness as biomarkers reflecting disease changes in biological fluids such as serum or synovial fluid (75). For instance, lubricin (PRG4), the only protein that was identified within the top ten in all knee compartments, was identified as a potential biomarker in human synovial fluid or the diagnosis of OA in a clinical observational study in 2022 (76). Furthermore, PRG4 injections have shown therapeutic potential in protecting against OA development by slowing the rate of cartilage breakdown in OA models (77). However, reported data regarding PRG4 remain controversial (78). Another example protein is the major articular cartilage aggrecanase, ADAMTS5, the inhibition of which was demonstrated as potentially useful for OA treatment in two very promising clinical trials (76).

**Table 1:** Modulation in OA of the top ten popular proteins identified in the different knee compartments by literature mining

Uniprot	Name	Location	Expression in OA	Description	Ref.
P08254	MMP3	Plasma	Up	MMP3 protein levels are higher in OA patients than in controls.	(82)
		Serum	Up	MMP3 protein levels in intermediate and advanced OA patients are higher than in early OA patients and healthy controls.	(83)
P03956	MMP1	Synovial fluid	Up	MMP1 protein levels are higher in OA patients than in controls.	(82, 84)
		Plasma	Up	MMP1 protein levels are higher in OA patients than in controls.	(82)
		Serum	Up	MMP-1 protein levels in intermediate and advanced OA patients are higher than in early OA patients and healthy controls.	(83)
Q92954	PRG4	Meniscus	Down	IHC analysis revealed strong lubricin immunostaining in normal menisci in contrast to OA menisci. Quantitative ELISA and Western blot analysis confirmed the above results.	(85)
		Synovial fluid	Down	PRG4 protein levels significantly decreased in the OA patients in comparison to healthy donors.	
		Plasma	Down	PRG4 protein levels significantly decreased in TJA patients compared to healthy donors.	(86)
P45452	MMP13	Synovial fluid	Up	Patients with KL grade II-III have higher protein levels of MMP13 than those with KL I.	(84)
P34810	CD68	Synovial membrane	Up	CD68 positive macrophages in the synovium significantly increased at 16 weeks in an OA mice model compared with control reference.	(87)
P01584	IL1B	Synovial fluid	Down	Patients with KL grade II-III-IV have lower protein levels of IL1B than those with KL I	(84)
		Serum	Up	IL-1B protein levels increased in patients with KOA compared with healthy controls. No differences when analysed independent KL stages.	(88)
		Plasma	Up	IL1B protein levels significantly increased in TJA patients compared to healthy donors.	(86)
Q16552	IL17A	Synovial fluid and serum	Up	SF IL-17A protein levels had strong positive correlations with radiographic severity. Serum levels of IL-17A were significantly higher in knee OA patients than controls.	(89)
P49747	COMP	Serum	Up	Longitudinal studies report higher serum COMP protein levels in patients who later demonstrate disease progression. Serum levels of COMP positive correlate with OA severity.	(73, 90)
O75173	ADAMTS4	Articular cartilage	Up	ADAMTS4 expression was higher in articular chondrocytes even in early-OA than controls.	(91)
P14555	PLA2G2A	Articular cartilage	Down	PLA2G2A is highly synthesized in control chondrocytes compared to OA.	(92)
P01033	TIMP1	Synovial fluid	Down	MMP1 levels are higher in patients with mild OA (KL grade I-II) than late-OA (KL III-IV)	(84)
Q9UNA0	ADAMTS5	Articular cartilage	Up	ADAMTS5 expression was higher in articular chondrocytes even in early-OA than controls.	(91)
		Serum	Up	ADAMTS5 protein levels are higher in intermediate and late-OA patients than in early-OA patients and healthy controls.	(83)
Q9HCJ1	ANKH	Meniscus	Up	ANKH expression was upregulated in OA meniscal cells compared to normal.	(93)
Q03692	COL10A1	Meniscus	Up	COL10A1 transcripts are higher in OA meniscus than control.	(94)
		Articular cartilage	Up	COL10A1 expression increased during OA progression. IHC confirmed the above results.	(95)
P02458	COL2A1	Articular cartilage	Down	Gradual decrease in the COL2A1 expression during cartilage degeneration in OA. IHC confirmed the above results. The expression of COL2A1 negatively correlated with OA.	(95)
Q9BXN1	ASPN	Blood	Up	ASPN protein levels increased in freshly prepared PBLs isolated from late knee OA patients compared to control.	(39)
		Articular cartilage	Up	ASP expression is higher in OA articular cartilage than control.	
Q9H1U4	MEGF9	Blood	Up	MEGF9 expression is higher in OA than control. IHC confirmed the above results.	(96)
E7EX88	ACAN	Articular cartilage	Down	Gradual decrease in the ACAN expression during cartilage degeneration in OA. The expression of ACAN negatively correlated with OA.	(95)
P20908	COL5A1	Articular cartilage	Up	COL5A1 is upregulated in OA damaged cartilage.	(97)
P58215	LOXL3	Articular cartilage	Up	LOXL3 higher protein levels in OA damaged cartilage than control.	(98)
P48436	SOX9	Articular cartilage	Up	SOX9 expression is higher in OA cartilage-derived cells compared to control. Significantly higher in mild and moderate OA than in severe OA-derived cells. SOX9 overexpression alleviates the progression of experimental OA.	(99, 100)
		Articular cartilage	Down	Gradual decrease in the SOX9 expression during cartilage degeneration in OA. The expression of *SOX9* negatively correlated with OA.	(95)

Abbreviations: IHC, immunohistochemistry; KL, Kellgren–Lawrence grading system; MSC, mesenchymal stem cell; OA, Osteoarthritis; PBLs, peripheral blood lymphocytes; SF, Synovial fluid; TJA, total joint arthroplasty.

On the other hand, the present approach also identified widely cited proteins in the knee that were not previously associated with OA in the literature but instead with other rheumatic diseases. This is the case of MEGF6 and Tesmin (TESMIN), which are associated with osteoporosis (79, 80). Thus, the strategy applied in this work identified various proteins that will require further study to determine their participation in the OA process. For example, the established role of Delta-like two in the downregulation of chondrogenesis suggests it is a potential target for the treatment of cartilage-related diseases such as OA (81).

## Conclusion

The present study provides a novel atlas of the knee based on the proteins present within the different knee tissues that are most cited in the literature. This work was aligned with the RAD-HPP goals in describing the proteomes of the human knee to assemble a prioritized list of proteins. The data collected here represent a highly valuable source of information on proteins that are clinically relevant in a complex and highly prevalent pathology such as OA. The application of literature-mining tools to analyses of a highly specialized organ, such as the knee joint, not only contributes to determining global trends through well-described proteins but also points out high-priority proteins for further targeted proteomics analyses to elucidate their roles in associated pathologies.

## Data Availability

This manuscript contains supplemental data. All data are contained within the manuscript and its Supplemental Data section.

## Supplemental data

This article contains supplemental data.

## Conflict of interest

We certify that there is no conflict of interest to disclose regarding the materials and data discussed in this manuscript. The contents of this manuscript have not been copyrighted or published previously.
