# Supplementary material for: An Atlas of the Knee Joint Proteins and Their Role in Osteoarthritis Defined by Literature Mining
Source: Mol Cell Proteomics. 2023 Jun 24;22(8):100606. doi: 10.1016/j.mcpro.2023.100606 (PMC10393810; doi:10.1016/j.mcpro.2023.100606)
Supplement: Supplemental Tables S1 and S2 and Figures S1–S4 [file mmc3.docx]

Supplemental Data for:

**An atlas of the knee joint proteins and their role in osteoarthritis defined by literature mining**

*Rocío Paz-González^1^, Lucía Lourido^1^, Valentina Calamia^1^, Patricia Fernández-Puente^2^, Patricia Quaranta^1^, Florencia Picchi^1^, Francisco J Blanco^1,2*^and Cristina Ruiz-Romero^1,3*^*

^1^ Grupo de Investigación de Reumatología (GIR) - Unidad de Proteómica, Instituto de Investigación Biomédica de A Coruña (INIBIC), Complexo Hospitalario Universitario de A Coruña (CHUAC), Sergas. C/As Xubias de Arriba 84, 15006, A Coruña, Spain.

^2^ Universidade da Coruña (UDC), Grupo de Investigación de Reumatología y Salud (GIR-S), Departamento de Fisioterapia, Medicina y Ciencias Biomédicas, Centro de investigaciones Avanzadas (CICA), 15008, A Coruña, Spain.

^3^ Centro de Investigación Biomédica en Red de Bioingeniería, Biomateriales y Nanomedicina (CIBER-BBN), Av. Monforte de Lemos, 3-5. Pabellón 11, 28029, Madrid, Spain.

* Correspondence:

Cristina Ruiz-Romero: cristina.ruiz.romero@sergas.es

Francisco J Blanco: fblagar@sergas.es

**Table of contents**

**Supplemental Tables**

**Supplemental Table S1.** Top 100 most cited proteins associated with OA in its four most common localizations (knee, hip, hand and spine). Provided as an Excel table. Uniprot: Protein Uniprot Accession number; GN: Gene Name; PN: Protein Name; FP: Protein Total Publication Count (adjusted); FPT: Protein Topic-Specific Publication Count (adjusted); WCD: Weighted Co-publication Distance; Z: Z score of WCD; P: P value of WCD.

**Supplemental Table S2.** Top 100 most cited proteins in the six different knee compartments that have been analyzed in this study. Provided as an Excel table.

**Supplemental Figures**

**Supplemental Figure S1.** Functional enrichment networks built with the top 100 most cited proteins in: A) Articular cartilage and B) Subchondral bone. Blue: Cartilage development; Red: Extracellular matrix organization; Green: Chondrocyte differentiation; Light blue: Ossification process.

**Supplemental Figure S2.** Results of the analysis of synovium and synovial fluid. Functional enrichment networks built with the top 100 most cited proteins in: A) Synovial membrane and B) Synovial fluid. Pink: Inflammatory response; Orange: Immune response; Dark green: Extracellular matrix disassembly. C) Venn diagram comparing the overlap between the top 100 proteins identified in synovial membrane and synovial fluid through PubPular v3.1.

**Supplemental Figure S3.** Functional enrichment networks built with the top 100 most cited proteins in: A) Meniscus and B) Cruciate ligament. Red: Extracellular matrix organization; Yellow: Skeletal system development.

**Supplemental Figure S4.** Venn diagram comparing the overlap between the top 100 proteins identified through literature mining in the six knee joint components studied in this work. AC, articular cartilage; SB, subchondral bone; SM, synovial membrane; SF, synovial fluid; M, meniscus and CL, cruciate ligament.


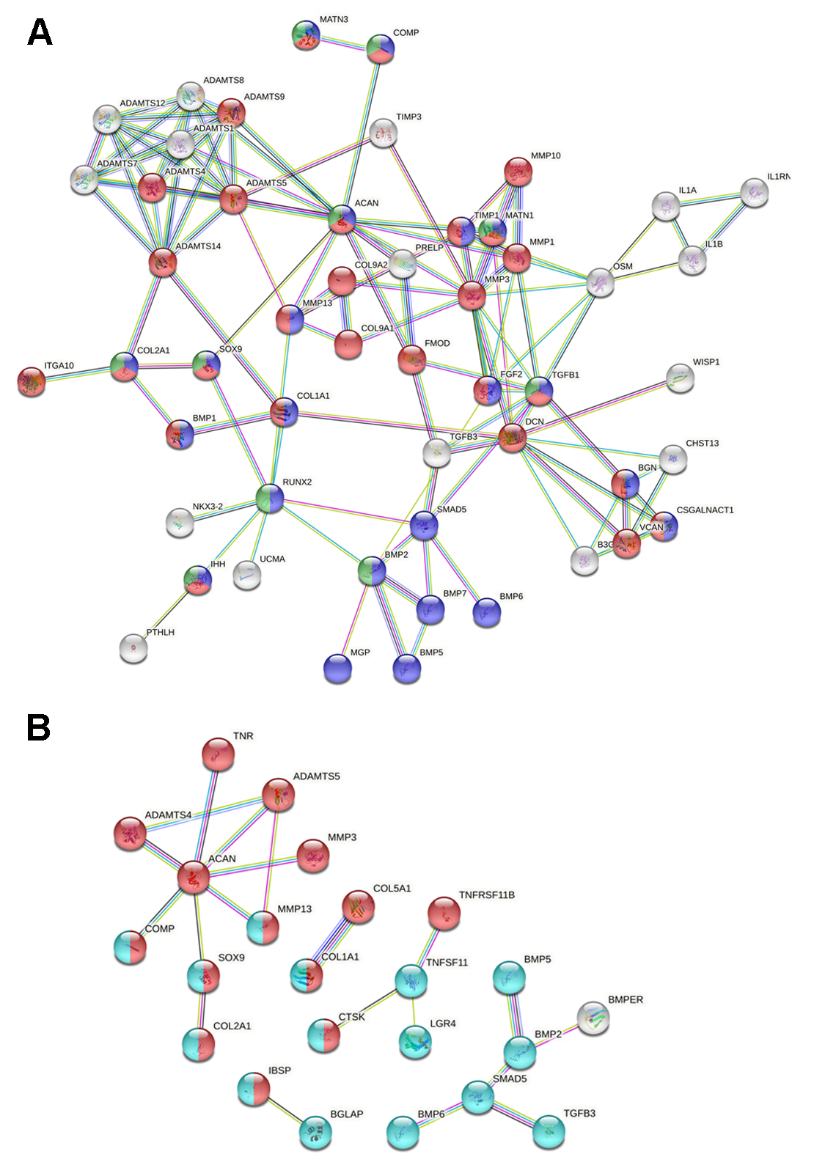


**Supplemental Figure S1.** Functional enrichment networks built with the top 100 most cited proteins in: A) Articular cartilage and B) Subchondral bone. Blue: Cartilage development; Red: Extracellular matrix organization; Green: Chondrocyte differentiation; Light blue: Ossification process.


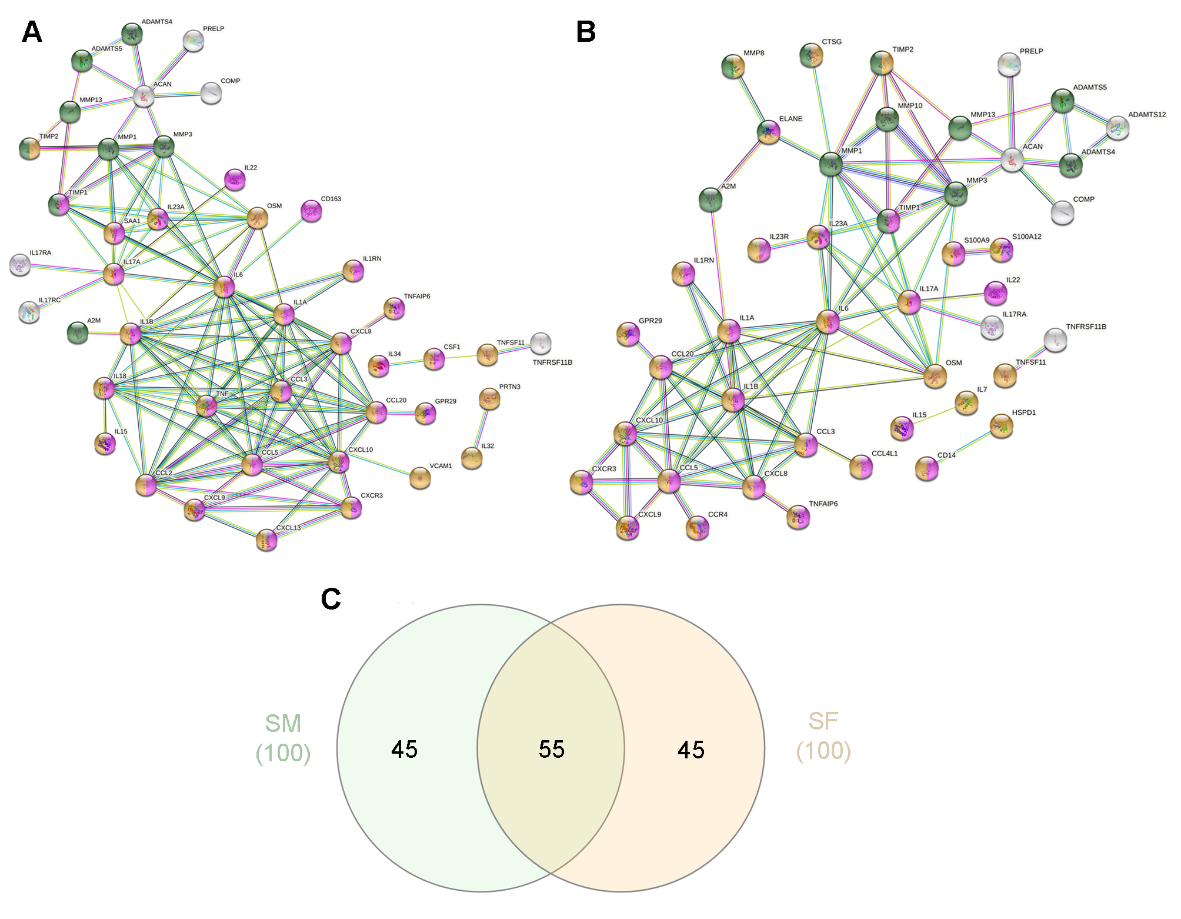


**Supplemental Figure S2.** Results of the analysis of synovium and synovial fluid. Functional enrichment networks built with the top 100 most cited proteins in: A) Synovial membrane and B) Synovial fluid. Pink: Inflammatory response; Orange: Immune response; Dark green: Extracellular matrix disassembly. C) Venn diagram comparing the overlap between the top 100 proteins identified in synovial membrane and synovial fluid through PubPular v3.1.


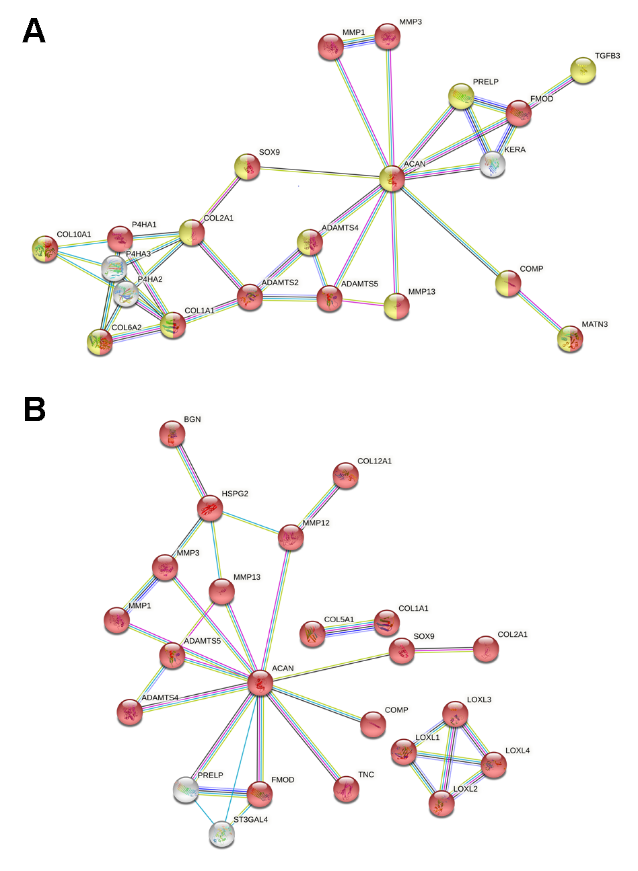


**Supplemental Figure S3.** Functional enrichment networks built with the top 100 most cited proteins in: A) Meniscus and B) Cruciate ligament. Red: Extracellular matrix organization; Yellow: Skeletal system development.


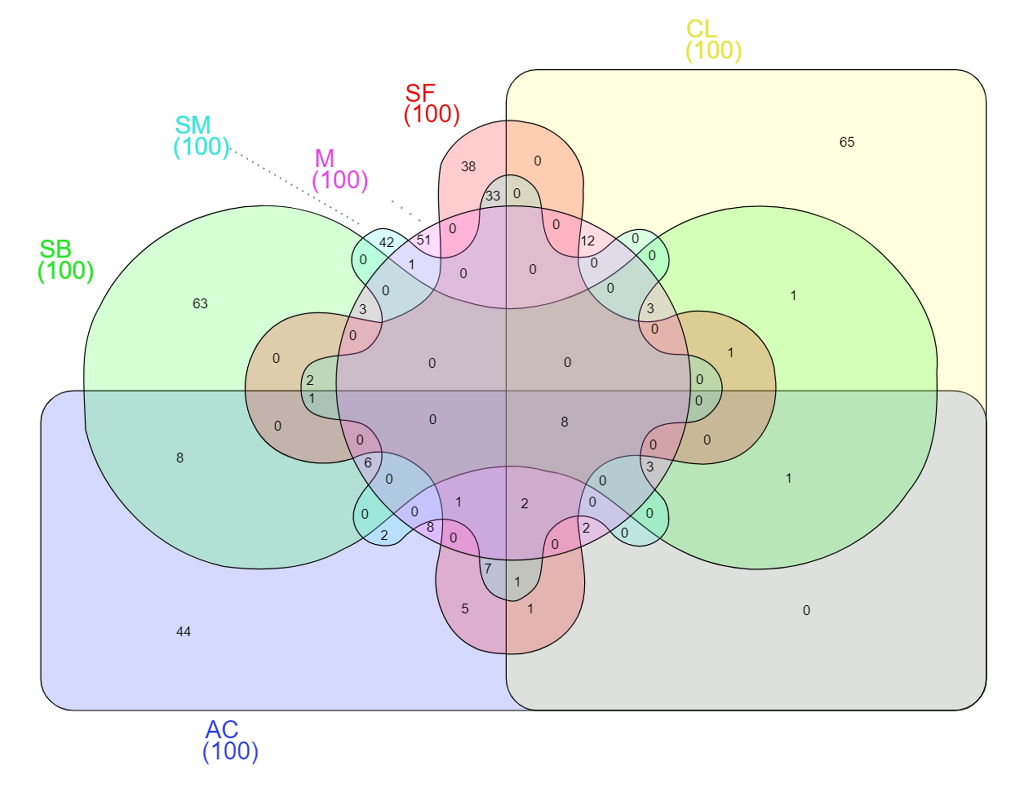


**Supplemental Figure S4.** Venn diagram comparing the overlap between the top 100 proteins identified through literature mining in the six knee joint components studied in this work. AC, articular cartilage; SB, subchondral bone; SM, synovial membrane; SF, synovial fluid; M, meniscus and CL, cruciate ligament.
